# A behavioral activation mobile application for depression among Korean young adults: a pilot study of multi-modal app usage patterns and clinical outcomes

**DOI:** 10.3389/fpsyt.2025.1707034

**Published:** 2026-01-22

**Authors:** Boyoung Kang, Minjee Jung, Ye-Seul Kim, Kyu-Man Han, Kee-Hong Choi

**Affiliations:** 1 ¹School of Psychology, Korea University, Seoul, Republic of Korea; 2 Department of Psychology, KU Mind Health Institute, Korea University, Seoul, Republic of Korea; 3 Department of Biomedical Sciences, Korea University College of Medicine, Seoul, Republic of Korea; 4 Department of Psychiatry, Korea University Anam Hospital, Korea University College of Medicine, Seoul, Republic of Korea

**Keywords:** behavioral activation, depression, digital therapeutics, feasibility, mobile app, mobile health

## Abstract

**Introduction:**

The incidence of depression is increasing among Korean youth; however, access to care remains limited due to systemic and cultural barriers. Behavioral activation (BA) is a first-line treatment for depression; nevertheless, few digital interventions have delivered its core components or addressed cultural needs. Accordingly, this study evaluated the preliminary feasibility, potential clinical benefits, engagement, and fidelity of the B-ACT, a culturally adapted digital BA intervention for Korean young adults with depression.

**Methods:**

In this single-arm seven-week pre-post study, 47 young adults with depressive disorders used the B-ACT app in hospital and community settings. This app delivered structured BA modules and activity tracking. Primary outcomes included changes in depressive symptoms, anxiety, the level of behavioral activation, and quality of life. Engagement metrics and positive activity data were also analyzed.

**Results:**

The participants showed significant reductions from baseline to post-treatment in depressive symptoms (BDI-II: -5.95, HDRS-17: -7.23, PHQ-8: -3.44) and anxiety (GAD-7: -2.47), along with increases in the level of behavioral activation (K-BADS: +11.50) and quality of life (SF-36 PCS: +6.62; MCS: +15.85). Post-treatment remission rates were 57.4% for HDRS-17, 27.7% for BDI-II, and 21.3% for PHQ-8, while response rates were 44.7%, 25.5%, and 21.3%, respectively. Greater participation in positive activities predicted a steeper decline in symptoms over time. Participants with more severe baseline depressive symptoms tended to show greater absolute reductions in symptom scores. The B-ACT showed high adherence and promising clinical change across hospital and community settings.

**Conclusions:**

The B-ACT shows promise as a scalable and culturally responsive intervention for depression among young Korean adults. Participants demonstrated significant improvements in depressive and anxiety symptoms, the level of behavioral activation, and quality of life from baseline to post-treatment. Involvement in activities that elicit positive emotions emerged as a key therapeutic mechanism. Randomized controlled trials are needed to evaluate long-term outcomes and broader applicability.

## 1 Introduction

Depression is the leading cause of disability worldwide, affecting an estimated 322 million people (1). The lifetime prevalence of major depressive disorder (MDD) is approximately 15% in most countries (2), and South Korea has reported a year-on-year rising trend in depression treatment (3). Beyond individual suffering, depression costs the global economy nearly USD 1 trillion annually in lost productivity (1), and is a major risk factor for suicide, accounting for a substantial proportion of suicide deaths (4, 5). Among young adults, depressive disorders often emerge during a critical developmental period marked by academic, vocational, and relational transitions. Depression during this period is associated with substantial functional impairment and elevated suicide risk (2, 4, 5).

Although conventional first-line antidepressant treatment is clinically effective, discontinuation reactions are common. Selective serotonin reuptake inhibitors (SSRIs) with shorter half-lives, such as paroxetine and fluvoxamine, are associated with a 50% discontinuation rate, often owing to side effects such as dizziness, nausea, and insomnia. Moreover, structural limitations of care provision remain problematic (5). A WHO report indicated that the Western Pacific Region, including South Korea, has only 15.4 mental health workers per 100,000 people (6). This figure is far below the high-income country average of 62.2 and indicates a significant workforce shortage despite South Korea’s economic standing.

Given these pharmacological and systemic constraints, there is increasing demand for scalable psychological treatments. Behavioral activation (BA), a treatment grounded in behavioral theory, has emerged as a promising approach. It helps individuals re-engage in meaningful value-driven activities while reducing avoidance behaviors that maintain depressive symptoms (7). A meta-analysis of 16 randomized trials with 780 participants reported a large acute effect size (g = 0.87) with long-term benefits comparable to those of full cognitive behavioral therapy (CBT) packages (8). In clinical practice, depressive disorders in adults are typically diagnosed using standardized systems such as the Diagnostic and Statistical Manual of Mental Disorders (DSM), and treatment decisions follow major pharmacological and psychotherapeutic guidelines. Within these frameworks, first-line options include antidepressant medication, structured psychotherapy, or their combination, depending on symptom severity and clinical history. Recent clinical guidelines, including those issued by the Canadian Network for Mood and Anxiety Treatments (CANMAT) and the National Institute for Health and Care Excellence (NICE), increasingly position BA as first-line psychotherapy, particularly for moderate-to-severe depressive symptoms (9).

However, most digital apps fail to implement the essential elements of BA. A review of 117 depression apps found that only 12 included CBT or BA content, with fewer than 20% incorporating core BA techniques such as activity monitoring and scheduling (10). This is a major gap, given BA’s effectiveness and empirical support. While many digital tools prioritize general psychoeducation or mindfulness, they often omit behaviorally targeted components that drive measurable change (10).

Recent systematic reviews and meta-analyses of internet-based psychological interventions, including web-delivered CBT and BA, have demonstrated clinically meaningful reductions in depressive symptoms and response rates comparable to face-to-face psychotherapy in adults diagnosed with MDD (11). However, these internet-based approaches largely depend on desktop or web-browser access and do not consistently incorporate real-time feedback, ecological momentary monitoring, or continuous activity-tracking features that may be essential for behavioral activation mechanisms.

Some widely used digital apps, such as Woebot and Wysa, have shown effectiveness and are often cited as accessible tools for emotional support and mental health education. Woebot demonstrated short-term symptom reduction in a two-week randomized controlled trial (12), and Wysa showed similar effects in a mixed-methods study (13). However, neither app is tailored to Korean users, nor are they available in the Korean language or health systems. Similarly, SPARX, a gamified CBT-based program for adolescents, has empirical support, but is inaccessible in South Korea. More recently developed apps, such as Spark and Moodivate, have applied BA principles in a fully digital format (14, 15). However, these remain largely untested or unavailable in the Korean context. These structural and contextual limitations highlight the need to examine mobile applications that can deliver behavioral activation in real time and within daily life contexts.

This issue is particularly acute in South Korea, where the burden of depression has significantly increased. A report from the NCMH indicated that the number of patients diagnosed with depression in 2021 rose to 933,481, an increase of 35.1% compared to 2017 (16). The surge was even more pronounced among younger populations, with a 127.1% increase in those in their 20s and 90.2% in teenagers. Despite these increases, mental health service utilization remains low. Only 12.1% of individuals diagnosed with mental disorders reported receiving professional help (16), indicating a significant treatment gap.

Cultural factors further compound these issues. The persistent stigma surrounding mental illness remains a primary barrier to seeking help in South Korea. Individuals often avoid treatment because of fear of social judgment or discrimination (17). In many Asian cultures, mental illness is viewed as not only a personal issue but also a threat to familial harmony (18–20). These cultural dynamics underscore the need for accessible, private, and stigma-free interventions.

This pilot study is the first to evaluate the B-ACT in young adults who meet the diagnostic criteria for depressive disorders. The primary aim of this study was to assess the impact of B-ACT on depressive symptoms, anxiety, the level of behavioral activation and health-related quality of life over a seven-week period. The sample consisted of DSM-diagnosed adults with varying depressive symptom severity, including individuals with and without ongoing psychological or pharmacological treatment. This study also tested the hypothesis that greater app usage, such as login frequency and activity completion, can predict symptom improvement. Although this was a single-arm trial, this study also evaluated whether B-ACT faithfully delivers core BA mechanisms and whether these mechanisms correlate with improvements in clinical outcomes. Additionally, this study explored whether treatment effects differ between primary care and counseling centers. Collectively, these features indicate that this work should be understood as a pilot study rather than a randomized controlled clinical trial. These findings will help to determine the feasibility of scalable BA-based digital interventions in low-resource environments.

## 2 Methods

### 2.1 B-ACT intervention (Overview)

B-ACT is a mobile behavioral activation intervention developed to deliver evidence-based BA components in a flexible and culturally appropriate digital format for Korean young adults. Unlike conventional BA apps focusing primarily on psychoeducation, B-ACT was designed to integrate activity monitoring, value-based goal setting, and reinforcement strategies within daily life through mobile delivery. The app incorporates culturally tailored examples and language and provides real-time behavioral prompts. Detailed modules and functions are presented in Section 2.4.

### 2.2 Study design

This single-arm, open-label pre-post study evaluated the preliminary effectiveness, feasibility, engagement, and fidelity of the B-ACT, a seven-week digital BA intervention, in young adults with depressive symptoms. This study was conducted between July 2024 and March 2025 in two settings: a university hospital psychiatric outpatient clinic and a community counseling center. The study was approved by two institutional review boards (IRB Nos. 2023AN0456 and KUIRB-2024-0031-08), and written informed consent was obtained from all participants prior to enrollment.

### 2.3 Participants

A total of 47 participants with MDD were enrolled in the study. Although the B-ACT application was originally developed for individuals aged 13–39 years, the present pilot study was *a priori* designed to enroll participants aged 18–39 years, and recruitment was therefore limited to this predefined age range. Participants were recruited from the outpatient psychiatric clinic of Korea University Anam Hospital (n = 25) and from two counseling centers in Seoul, South Korea—the Korea University KU Mind Health Institute and the Mindeep CBT Center (n = 22)—through online advertisements and printed recruitment materials posted at the counseling centers. These sites provide psychiatric and psychological services to individuals seeking treatment or counseling. Individuals who expressed interest received study information and voluntarily chose to participate. Eligible individuals met the DSM-5 diagnostic criteria for MDD—including single or recurrent episodes or persistent depressive disorder—based on clinical interviews conducted by a board-certified psychiatrist at the hospital site or by two doctoral-level trainees in clinical and counseling psychology under the supervision of a licensed clinical psychologist at the counseling sites. Symptom severity ranged from mild to severe, as indicated by a Beck’s Depression Inventory-II (BDI-II) score ≥14. The inclusion criteria were as follows (1): age between 18 and 39 years (2), proficiency in the Korean language (3), ability to use a mobile device independently, and (4) for participants currently receiving psychiatric medication, a stable dose for at least four weeks prior to enrollment and throughout the digital BA intervention. Importantly, ongoing medication use was not required for study participation. Concomitant use of psychiatric medications was permitted only under specific conditions. Antidepressants were allowed only if the type and dosage remained unchanged. Antipsychotics were permitted only when prescribed as adjunctive treatment for depression, with both type and dosage kept stable. Benzodiazepines were permitted only at a stable pre-existing dose, and initiation of new prescriptions was prohibited. Hypnotics were limited to zolpidem or zopiclone when clinically indicated, with no dosage changes permitted. No other medication adjustments were allowed during the study period. These medications were permitted to reflect real-world clinical practice and contemporary treatment guidelines, in which antidepressants are standard first-line treatments for MDD, antipsychotics are commonly used as adjunctive agents, and benzodiazepines and hypnotics are frequently prescribed to manage comorbid anxiety and sleep-related symptoms (21, 22). All inclusion and exclusion criteria were applied at the screening visit through clinical interviews and, when available, review of medical records. The exclusion criteria were as follows (1): current or past diagnosis of bipolar disorder, psychotic disorder, or substance use disorder (2); active self-harm or active suicidal ideation (3); severe neurological or medical conditions that could interfere with study participation, such as epilepsy, brain tumors, or cerebrovascular disease; and (4) difficulties in understanding or completing the digital intervention.

### 2.4 Intervention: The B-ACT app

#### 2.4.1 Module structure and features

The B-ACT mobile application (developed by ROWAN Inc.) is a seven-week digital therapeutic program designed by a multidisciplinary team in psychiatry, clinical psychology, and software development to deliver structured BA therapy. The B-ACT guides users through weekly modules targeting the following key BA components: psychoeducation, mood-activity monitoring, value-based goal setting, reinforcement planning, problem-solving, mindfulness, and relapse prevention. The content of the seven weekly modules is detailed in **Supplementary Table S1**. Each module builds on the content of the previous week, and is anchored by psychoeducational videos that must be completed sequentially. Notably, the psychoeducational materials were culturally adapted for Korean young adults, incorporating examples of salient stressors in this population, such as academic performance decline and conflicts with parents, to enhance relevance and engagement. Access to the next module is time-based and unlocked weekly over the intervention period.

#### 2.4.2 Activity logging and emotion tracking

Users record their daily activities using a dedicated input screen with quick-access buttons for five core routines: sleeping, meals, personal hygiene, medication intake, and going out. They then select one of eight mood states: happiness, excitement, satisfaction, calmness, anger, anxiety, boredom, or sadness. The catalog of 441 activities was adapted from a prior BA mobile app (23), originally based on the Pleasant Events Schedule (24). For this study, the activity catalog was translated, culturally adapted, and expanded for Korean users by removing culturally irrelevant items, simplifying the language, and splitting compound entries. For example, “playing cricket” was replaced with “playing baseball,” “exploring with a metal detector” was adapted to “exploring new restaurants,” and “watching home videos” was updated to “using Over-The-Top (OTT) streaming services,” reflecting cultural relevance and contemporary leisure trends in Korea. To capture users’ affective states, mood ratings were based on the Pick-A-Mood framework, which reflects the circumplex model of affect (25, 26). Users can also manually input activities that are not included in the catalog, allowing personalized and flexible behavior tracking. Mood-activity entries are visualized on a calendar, enabling users to observe their emotional and behavioral patterns over time.

#### 2.4.3 Value alignment and behavioral statistics

The statistics tab provides weekly summaries of Patient Health Questionnaire-8 (PHQ-8) depression scores, as well as the frequency and system-assigned valence scores of positively valenced activities based on Korean affective norm ratings (27). The app also categorizes activities by emotional valence, helping users to identify mood-behavior patterns. To support value–behavior alignment, a radar chart is used to visualize the distribution of user activities across six life domains: interpersonal relationships, education and career, hobbies and leisure, health and religion, daily activities, and self-care. This distribution is compared with users’ self-reported value importance. These domains were synthesized and adapted from established BA frameworks and digital applications (23, 28, 29). Each of the 441 activities was pre-categorized into one of six domains to enable real-time mapping of behavior to personal values. Manually added activities were also assigned to one of these domains by users at the time of entry.

#### 2.4.4 Motivational strategies and user engagement

**Figure 1:**
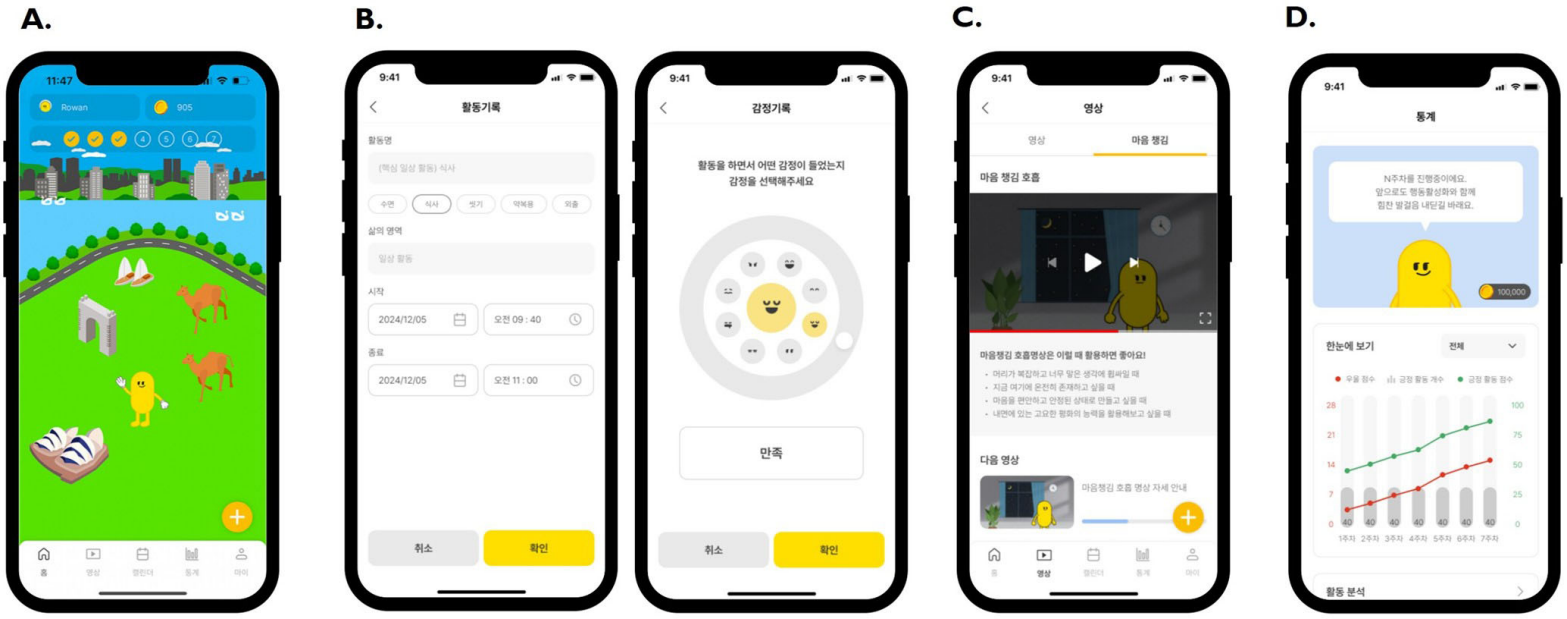
Representative screenshots of the B-ACT app illustrating core features and user interface. **(A)** Home screen with gamified elements, including avatar customization and interactive landscapes. **(B)** Activity and emotion logging screens: users select activities and tag associated emotions from predefined options. **(C)** Psychoeducational video interface displaying weekly therapeutic content. **(D)** Statistics dashboard visualizing weekly trends in positive activity frequency, system-assigned positive activity scores, and PHQ-8 depression scores. PHQ-8, Patient Health Questionnaire-8.

Gamified features were implemented to enhance engagement, including coin rewards, avatar customization, and home screen decoration. Coins were earned by logging activities with associated emotions or watching psychoeducational videos. In Week 3, users completed a value clarification task by rating the importance of six valued life domains, which served as a foundation for value–behavior alignment throughout the intervention. They then set personalized “missions” by defining value-based goals and rating them on expectancy, importance, and difficulty. Mindfulness videos were introduced in Week 6. In Week 7, users received a personalized report (“My Journey”) summarizing their behavioral trends and value–behavior alignment. Representative screenshots of the app are shown in **Figure 1**.

### 2.5 Measures

#### 2.5.1 Depressive symptoms

Depressive symptoms were evaluated using three validated instruments: the BDI-II, 17-item Hamilton Depression Rating Scale (HDRS-17), and PHQ-8. The BDI-II includes 21 items, each of which is scored on a 0–3 scale (total 0–63), with severity categorized as minimal (0–13), mild (14–19), moderate (20–28), and severe (29–63) (30). It demonstrates excellent reliability in both clinical and nonclinical populations (Cronbach’s α = 0.93) (30); the Korean version also shows high internal consistency (Cronbach’s α = 0.89) (31). The PHQ-8 consists of 8 items derived from the PHQ-9 (32), excluding the suicide-related item. This item was omitted for ethical reasons, as real-time monitoring of suicide risk and immediate clinical intervention could not be ensured during app-based, self-administered weekly assessments between in-person visits. Items are rated on a 0–3 Likert scale, yielding total scores from 0 to 24. Severity cutoffs are defined as none (0–4), subthreshold (5–9), mild (10–14), moderate (15–19), and severe (20–24). The scale shows comparable diagnostic performance to the PHQ-9 (33), and its validity has been confirmed in Korean clinical settings (34). The HDRS-17 is a clinician-administered instrument that assesses depressive symptoms over the past week (35). It includes 17 items that are rated on mixed 3- and 5-point scales, with total scores ranging from 0 to 52. Severity levels are typically classified as remission (0–7), mild (8–16), moderate (17–23), and severe (≥24) (36). The scale demonstrates strong inter-rater and test–retest reliability (37), and the Korean version shows acceptable internal consistency (Cronbach’s α = 0.76) (38). Treatment response on these depression measures was defined as a ≥50% reduction from baseline scores (39–41).

#### 2.5.2 Behavioral activation

The level of behavioral activation was measured using the Korean version of the Behavioral Activation for Depression Scale (K-BADS), a 25-item self-report instrument that evaluates core behavioral processes in depression (42). It comprises four subscales: Activation, Avoidance/Rumination, Work/School Impairment, and Social Impairment. Higher total scores, computed by reverse coding the non-activation subscales and summing them with the Activation score, indicate a higher level of behavioral activation. The scale has demonstrated high internal consistency (Cronbach’s α = 0.79–0.87) (43), with the Korean version also showing strong reliability (Cronbach’s α = 0.84).

#### 2.5.3 Anxiety symptoms

Anxiety symptoms were assessed using the Generalized Anxiety Disorder–7 (GAD-7), a 7-item self-report scale that measures anxiety severity over the past two weeks (44). Each item is rated from 0 to 3 (total score range: 0–21), with severity categorized as minimal (0–4), mild (5–9), moderate (10–14), or severe (15–21). The GAD-7 shows excellent internal consistency (α = 0.92), and its Korean version demonstrates comparable reliability (Cronbach’s α = 0.93) (45).

#### 2.5.4 Quality of life

Health-related quality of life was measured using the 36-Item Short Form Health Survey (SF-36), a widely validated self-report instrument (46). The scale comprises 36 items across eight subscales: physical functioning, role limitations due to physical health, role limitations due to emotional problems, bodily pain, social functioning, mental health, vitality, and general health perceptions. One additional item measures perceived health transition over the past year. These eight subscales are also used to compute two composite scores: the Physical Component Summary (PCS), which includes physical functioning, role-physical, bodily pain, and general health perceptions; and the Mental Component Summary (MCS), comprising vitality, social functioning, role-emotional, and mental health. Subscale scores are standardized from 0 to 100, with higher scores indicating better perceived health (47). The Korean version of the SF-36 has demonstrated excellent internal consistency (Cronbach’s α = 0.93–0.94) (48).

#### 2.5.5 App satisfaction

User experience was assessed using the User Version of the Mobile Application Rating Scale (uMARS), a 20-item self-report measure for evaluating mobile health app quality (49). It comprises four objective sub-domains (Engagement, Functionality, Aesthetics, and Information Quality) and a subjective quality domain. A 6-item impact subscale evaluates perceived effects across areas such as awareness, behavior, and help-seeking. All items are rated on a 5-point Likert scale, with higher scores indicating better quality. The impact subscale is analyzed separately. The scale shows strong internal consistency (Cronbach’s α = 0.90). As no validated Korean version exists, the uMARS was translated by a bilingual psychologist and reviewed by a multidisciplinary team for cultural and contextual validity.

#### 2.5.6 App usage metrics

Four metrics were extracted to quantify behavioral interactions during the seven-week intervention: total log count (app access frequency), total activity count (all logged activities), total positive activity (number of emotionally positive activities), and total positive activity score (sum of valence scores). Positive activity variables therefore represent the frequency and magnitude of engagement in emotionally positive activities. Valence scores, based on Korean affective norms (27), were assigned to positive emotions such as excitement, happiness, calmness, and satisfaction to compute a valence-weighted index of participation in emotionally positive activities. Positive activities were operationally defined as those accompanied by these positive affective states, and weekly valence-weighted positive activity scores were calculated by summing the valence values of all logged positive activities. Metrics were analyzed weekly (Weeks 1–7) and cumulatively.

### 2.6 Procedure

The study consisted of nine scheduled visits: Visit 1 (screening), Visits 2–8 (seven-week intervention), and Visit 9 (post-treatment follow-up). All study visits were conducted as research-dedicated visits and were independent of participants’ routine clinical care. At the hospital site, eligibility screening (Visit 1) was conducted in person. At the counseling sites, eligibility screening (Visit 1) was conducted entirely online for all participants. Regardless of recruitment source, all participants were required to attend in-person visits at the study site for the major assessment sessions at Visit 2 (baseline, Week 1), Visit 5 (midpoint, Week 4), and Visit 9 (post-treatment, one week after the intervention). These in-person visits were required specifically for clinician-administered assessments (HDRS-17) and to ensure adequate structured safety monitoring. The remaining interim visits (Visits 3–4 and 6–8) focused on adherence and safety monitoring and were conducted either in person or by phone, depending on visit type, participant availability, and preference. At the hospital site, clinician-administered assessments, including the HDRS-17, were conducted directly by a board-certified psychiatrist, who was also responsible for diagnostic confirmation and overall clinical oversight. A trained research nurse assisted with study procedures and administered self-report questionnaires under the psychiatrist’s supervision. At the counseling sites, all assessments, including clinician-administered scales, were conducted by two doctoral-level trainees in clinical and counseling psychology under the supervision of a licensed clinical psychologist. Prior to study initiation, these doctoral-level assessors received standardized training in the administration of all clinical rating scales. These assessors were independent of participants’ clinical treatment at the counseling sites, as participants were newly referred for study participation and had no prior therapeutic relationship with the assessors at those sites at the time of enrollment. All assessments were performed using standardized research procedures. At Visit 1 (screening), clinical history and psychiatric comorbidities were assessed through clinical interviews, with additional verification by medical record review when available. Participants also completed the BDI-II as part of the eligibility assessment. At Visit 2 (baseline, Week 1), participants completed a clinician-administered assessment of depressive symptoms (HDRS-17) and self-report questionnaires assessing depressive symptoms (BDI-II), behavioral activation (K-BADS), anxiety (GAD-7), and quality of life (SF-36). The participants completed these self-report assessments in paper format under assessor supervision. The PHQ-8 was collected exclusively via the mobile app during the intervention period. Immediately after the baseline visit, participants were onboarded to the intervention by trained staff, marking the start of app use. Each treatment week began with an automated in-app PHQ-8, completed independently by participants. At Visit 5 (midpoint, Week 4), participants repeated the full baseline assessment battery on paper; the PHQ-8 was not duplicated at this time, as it had already been collected via the app. At Visit 9 (post-treatment), participants completed the final assessment battery in person, including the PHQ-8 on paper, as app use had ended. Participants also provided qualitative feedback at this stage. Across all sites, standardized safety monitoring procedures and referral resources were consistently maintained throughout the study.

### 2.7 Statistical analyses

All statistical analyses were conducted using IBM SPSS Statistics (version 27 and 29 for Mac; IBM Corp., 2023) and R software (version 4.3.2; R Core Team). Descriptive statistics and frequency analyses were performed to examine participants’ demographic characteristics as well as app usage patterns, including weekly log-in frequency, activity counts, and positive activity measures. To assess group differences at baseline between the hospital outpatient and community counseling center settings, independent-samples t-tests were conducted for the demographic and clinical variables, and chi-square tests were used for categorical variables.

Before conducting the multilevel analyses, we calculated descriptive statistics and pre–post change scores for all outcome measures (BDI-II, HDRS-17, PHQ-8, K-BADS, GAD-7, and the SF-36 PCS and MCS). Subsequently, two-level multilevel modeling (MLM) was employed to examine the effects of the B-ACT intervention on depression and related mental health outcomes. In these models, Level 1 represented within-subject changes over time (pre-, mid-, and post-intervention), and Level 2 represented between-subject differences across individuals. MLM allows for the inclusion of all participants regardless of missing data during the intervention period and is particularly well suited for longitudinal data, as it enables the simultaneous modeling of intra-individual and inter-individual changes (50, 51).

Effect sizes were calculated using Cohen’s *d* for key outcomes to support the interpretation of the impact of the intervention. To examine the effect of positive activity participation on depressive symptoms over time, we conducted an additional multilevel model using time as a continuous Level 1 predictor with weekly PHQ-8 scores as an outcome. In this model, Level 1 represented within-subject variation across weekly measurements (Weeks 1–7), and Level 2 represented between-subject differences across individuals. The model included two interaction terms (Weekly positive activities × Time, Weekly log counts × Time) and covariates such as age, sex, and current use of psychotropic medication.

Additional analyses were conducted to examine the relationship between usage of the app and clinical outcomes. First, Pearson’s correlation analyses were conducted to assess the relationship between weekly changes in PHQ-8 scores and positive activity variables. Correlations between changes in PHQ-8, BDI-II, and HDRS-17 scores from baseline to post-treatment and app usage variables were also assessed. Simple and multiple regression analyses were performed to assess the relationship between changes in BDI-II and HDRS-17 scores and app usage variables, respectively. Additionally, to evaluate the relationship between app satisfaction (uMARS) and clinical results, correlation analyses were performed between uMARS scores and changes in PHQ-8, BDI-II, and HDRS-17 scores as well as app usage variables.

Furthermore, as a supplementary descriptive analysis, we compared the mean changes in PHQ-8, BDI-II, and HDRS-17 scores across the three time points (baseline, 4 weeks, and 8 weeks) within the depression severity group (normal, mild, moderate, and severe) using repeated measures analysis of variance (ANOVA). The severity group cutoffs corresponding to normal, mild, moderate, and severe were as follows: PHQ-8 (≤9, 10–14, 15–19, ≥20), BDI-II (≤13, 14–19, 20–28, ≥29), and HDRS-17 (≤7, 8–16, 17–23, ≥24). To compare the mean changes in PHQ-8, HDRS-17, and BDI-II scores between hospital outpatient and community counseling center settings, independent-samples t tests were conducted, and the effect size was calculated using Cohen’s *d*. All statistical tests were two-tailed, and the significance level was set at *p* <.05.

All statistical assumptions were checked prior to analysis, including normality of residuals, linearity, and multicollinearity. When assumptions were violated, robust estimators or Greenhouse–Geisser corrections were applied. For the multilevel models, normality was examined using Q–Q plots of Level 1 residuals and Level 2 random intercepts, and linearity was assessed using residual-versus-fitted value plots. Multicollinearity was assessed using variance inflation factors (VIFs) and grand-mean centering was applied to continuous predictors to minimize collinearity. All available data were included in the multilevel analyses using restricted maximum likelihood estimation. The residuals followed an approximately normal distribution, as indicated by the linear pattern of the Q–Q plots. In addition, the residuals were randomly scattered around the reference line in the residual-versus-fitted value plots, confirming that the assumption of linearity was met. All VIF values were below 10, indicating no serious multicollinearity among the predictors.

## 3 Results

### 3.1 Participant characteristics

A total of 47 participants were enrolled in this study. Following the baseline assessment, five participants discontinued (three from the hospital outpatient setting and two from the community counseling center): three due to changes in psychotropic medication, one due to voluntary withdrawal, and one due to non-completion of the mid-intervention assessment. Consequently, 42 participants completed the mid-intervention assessment, yielding a retention rate of 89.4%. An additional participant from the hospital group withdrew following the mid-assessment owing to medication adjustment, resulting in 41 participants completing the full intervention, corresponding to an overall retention rate of 87.2%. The participant characteristics are presented in **Table 1**. Apart from current medication use, no significant baseline differences were observed between the hospital outpatient and community counseling center groups in demographic or clinical variables (all ps ≥.08). Current psychotropic medication use was significantly more frequent in the hospital outpatient group than in the community counseling center group (100% vs. 45.5%; *χ²* (1) = 18.31, *p* <.001; **Table 1**). The mean age of participants was 26.60 years (SD = 5.09); specifically, the mean age was 27.16 (SD = 5.31) in the hospital group and 25.95 (SD = 4.87) in the community counseling center group. A greater proportion of participants were female (n = 28, 59.6%) than male (n = 19, 40.4%). Regarding educational attainment, most participants had completed a college degree (n = 22, 46.8%), followed by high school graduates (n = 20, 42.6%). At baseline, the average depression severity score indicated mild-to-moderate symptoms; the mean BDI-II score was 25.49, the mean HDRS-17 score was 14.57, and the mean PHQ-8 score was 11.81. Most participants (n = 35, 74.5%) were currently taking psychotropic medication. Detailed information on psychotropic medication use according to setting is shown in **Supplementary Table S2**. No serious adverse events or safety concerns requiring urgent clinical intervention were observed during the study period.

**Table 1:** Descriptive statistics on demographic variables at baseline.

Variable	All (n=47)	Hospital outpatient (n=25)	Community counseling center (n=22)	Test statistic
Age	26.60 (5.09)	27.16 (5.31)	25.95 (4.87)	*t* (45) = .81, *p* = .42
Sex				χ2 (1) = 2.97, *p* = .08
Male	19 (40.4%)	13 (52.0%)	6 (27.3%)	
Female	28 (59.6%)	12 (48.0%)	16 (72.7%)	
Educational level				χ2 (3) = 5.01, *p* = .17
Middle school graduate	1 (2.1%)	1 (4.0%)	0 (0.0%)	
High school graduate	20 (42.6%)	10 (40.0%)	10 (45.5%)	
College graduate	22 (46.8%)	10 (40.0%)	12 (54.5%)	
Enrolled in a graduate program	4 (8.5%)	4 (16.0%)	0 (0.0%)	
Baseline depression				
BDI-II	25.49 (9.36)	25.80 (10.15)	25.14 (8.59)	*t*(45) = .24, *p* = .81
HDRS-17	14.57 (3.91)	15.48 (4.05)	13.55 (3.56)	*t*(45) = 1.74, *p* = .09
PHQ-8	11.81 (4.55)	11.44 (4.67)	12.23 (4.47)	*t*(45) = -.59, *p* = .56
Other baseline measures				
K-BADS	70.62 (22.14)	75.16 (22.17)	65.45 (21.43)	*t*(45) = 1.521, *p* = .14
GAD-7	8.98 (4.62)	8.64 (4.08)	9.36 (5.24)	*t*(45) = -.531, *p* = .60
SF-36 (PCS)	59.71 (21.67)	56.74 (19.89)	63.09 (23.53)	*t*(45) = -1.00, *p* = .32
SF-36 (MCS)	51.25 (21.20)	51.17 (20.70)	51.33 (22.24)	*t*(45) = -.03, *p* = .98
Current medication				χ2 (1) = 18.31, *p* < .001
Yes	35 (74.5%)	25 (100%)	10 (45.5%)	
No	12 (25.5%)	0 (0%)	12 (54.5%)	

Data are presented as mean ± standard deviation for age, BDI-II scores, HDRS-17 scores, PHQ-8 scores, K-BADS scores, GAD-7 scores, and SF-36 scores.

*p*-values for comparisons of age, BDI-II scores, HDRS-17 scores, PHQ-8 scores, K-BADS scores, GAD-7 scores, and SF-36 scores between hospital outpatient and community counseling center were obtained using independent samples t-tests. *p*-values for the distribution of sex, education level, and current medication are obtained using chi-squared tests.

BDI-II, Beck Depression Inventory-II; HDRS-17, 17-item Hamilton Depression Rating Scale; PHQ-8, Patient Health Questionnaire-8; K-BADS, Korean version of the Behavioral Activation for Depression Scale; GAD-7, Generalized Anxiety Disorder–7; SF-36, 36-Item Short Form Health Survey; PCS, Physical Component Summary; MCS, Mental Component Summary.

### 3.2 App usage patterns

Participants demonstrated consistent engagement with the B-ACT app throughout the seven-week intervention. On average, participants logged into the app 5.67 times per week (SD = 1.44), with weekly log-ins ranging from 1.43 to 7.00. The mean number of activities recorded per week was 27.73 (SD = 19.44), with a range of 5.29 to 80.14. Participants performed an average of 19.33 positive activities per week (SD = 14.41; range: 1–62). In addition, the mean weekly positive activity score based on in-app valence ratings was 108.60 (SD = 81.11), with values ranging from 7.10 to 348.78.

### 3.3 The effects of B-ACT

The mean BDI-II score decreased from 25.49 at baseline to 19.54 at post-treatment (-5.95 points), the HDRS-17 score declined from 14.57 to 7.34 (-7.23 points), and the PHQ-8 score decreased from 11.81 to 8.37 (-3.44 points), indicating overall reductions in depressive symptoms. Other outcome variables also showed improvements, with the mean K-BADS score increasing from 70.62 to 82.12 (+11.50 points), the GAD-7 score decreasing from 8.98 to 6.51 (-2.47 points), the SF-36 PCS score increasing from 59.71 to 66.33 (+6.62 points), and the SF-36 MCS score increasing from 51.25 to 67.10 (+15.85 points) from baseline to post-treatment. For detailed results, see **Table 2**.

**Table 2:** Descriptive statistics, results of multilevel analysis, and effect sizes (Cohen’s *d*).

Measure	Descriptive statistics *M*(SD)			Multilevel analysis				Cohen’s *d*
	Baseline	Mid-treatment	Post-treatment	Effects estimate	SE	*t*	*p*	
BDI-II	25.49 (9.360)	21.71 (10.449)	19.54 (12.040)	–	–	–	–	0.474
Intercept	–	–	–	25.975	7.418	3.502	.001^**^	
Time	–	–	–	-2.604	0.794	-3.282	.002^**^	
HDRS-17	14.57 (3.911)	10.69 (4.906)	7.34 (4.151)	–	–	–	–	1.898
Intercept	–	–	–	21.397	3.165	6.760	< .001^***^	
Time	–	–	–	-3.544	0.300	-11.813	< .001^***^	
PHQ-8	11.81 (4.548)	9.25 (4.970)	8.37 (5.526)	–	–	–	–	0.736
Intercept	–	–	–	12.809	3.510	3.650	< .001^***^	
Time	–	–	–	-1.707	0.350	-4.867	< .001^***^	
K-BADS Total	70.62 (22.136)	75.79 (20.891)	82.12 (24.422)	–	–	–	–	0.475
Intercept	–	–	–	73.929	15.414	4.796	< .001^***^	
Time	–	–	–	5.555	1.706	3.257	.002^**^	
K-BADS Activation subscale	14.06 (7.450)	15.50 (6.837)	17.44 (7.553)	–	–	–	–	0.406
Intercept	–	–	–	16.031	5.384	2.977	.005^**^	
Time	–	–	–	1.593	0.581	2.740	.009^**^	
K-BADS Work/School impairment subscale	16.15 (6.676)	13.69 (6.241)	12.02 (6.319)	–	–	–	–	0.600
Intercept	–	–	–	22.954	4.325	5.307	< .001^***^	
Time	–	–	–	-2.010	0.529	-3.969	< .001^***^	
GAD-7	8.98 (4.623)	8.48 (5.597)	6.51 (5.060)	–	–	–	–	0.503
Intercept	–	–	–	10.158	3.638	2.792	.008^**^	
Time	–	–	–	-1.183	0.358	-3.305	.002^**^	
SF-36 (PCS)	59.71 (21.665)	62.11 (21.396)	66.32 (22.444)	–	–	–	–	0.264
Intercept	–	–	–	46.876	16.372	2.863	.006^**^	
Time	–	–	–	2.752	1.449	1.900	.064	
SF-36 (MCS)	51.25 (21.195)	55.42 (23.521)	67.10 (28.524)	–	–	–	–	0.575
Intercept	–	–	–	45.731	16.097	2.841	.007^**^	
Time	–	–	–	7.736	2.010	3.849	< .001^***^	

*M*, mean; SD, Standard deviation; SE, Standard error; *t*, *t*-value; ^*^p<.05, ^**^p<.01, ^***^p<.001.

BDI-II, Beck Depression Inventory-II; HDRS-17, 17-item Hamilton Depression Rating Scale; PHQ-8, Patient Health Questionnaire-8; K-BADS, Korean version of the Behavioral Activation for Depression Scale; GAD-7, Generalized Anxiety Disorder–7; SF-36, 36-Item Short Form Health Survey; PCS, Physical Component Summary; MCS, Mental Component Summary.

A multilevel analysis was conducted to evaluate how the clinical outcomes changed across the three assessment points: baseline (Week 0), mid-intervention (Week 4), and post-intervention (Week 8). Time was modeled as a continuous variable, with the baseline measurement serving as the reference level. Age, gender, and current medication use were included as covariates in all models. Each model included time as a fixed effect, with random intercepts and slopes for participants.

Analyses revealed statistically significant changes across all major outcome variables. Depression symptoms showed meaningful improvement over time. Compared to baseline, BDI-II scores were significantly reduced by post-intervention (B = -2.604, *p* = .002), as were PHQ-8 (B = -1.707, *p* <.001) and HDRS-17 (B = -3.544, *p* <.001), with effect sizes ranging from small to large (*d* = 0.474–1.898). BA levels significantly increased over time. Total K-BADS scores increased from baseline to post-intervention (B = 5.555, *p* = .002), with a small effect (*d* = 0.475). Among its subcomponents, activation scores showed a significant increase (B = 1.593, *p* = .009), while the work/school impairment subscale showed significant decrease (B = -2.100, *p* <.001). Anxiety symptoms, measured by GAD-7, decreased significantly from baseline to post-intervention (B = -1.183, *p* = .002), with a medium effect (*d* = 0.503). Additionally, quality of life assessed by the MCS of the SF-36 significantly improved over time (B = 7.736, *p* <.001) with a medium effect (*d* = 0.575). **Table 2** presents the effect sizes (Cohen’s *d*) and results of the multilevel analysis.

In addition to continuous symptom changes, remission and response rates were assessed post-intervention (Week 8). The remission rate was 27.7% for BDI-II (95% CI, 16.9–41.8%), 57.4% for HDRS-17 (95% CI, 43.3–70.5%), 21.3% for PHQ-8 (95% CI, 12.0–34.9%), and 34.0% for GAD-7 (95% CI, 22.2–48.3%). The response rate was 25.5% for BDI-II (95% CI, 15.3–39.5%), 44.7% for HDRS-17 (95% CI, 31.4–58.8%), 21.3% for PHQ-8 (95% CI, 12.0–34.9%), and 31.9% for GAD-7 (95% CI, 20.4–46.2%).

### 3.4 Weekly changes in PHQ-8 scores and positive activities

The relationships between weekly changes in PHQ-8 scores and the number of positive activities were examined. Pearson’s correlation analyses revealed no statistically significant correlations between changes in PHQ-8 scores and the number of positive activities performed (see **Supplementary Table S3**). This finding indicates that the weekly number of positive activities was not significantly associated with changes in depression symptoms. Similarly, no consistent significant correlations were observed between changes in PHQ-8 scores and changes in the number of positive activities (see **Supplementary Table S4**).

### 3.5 Effects of positive activity participation and app usage on depression

**Figure 2:**
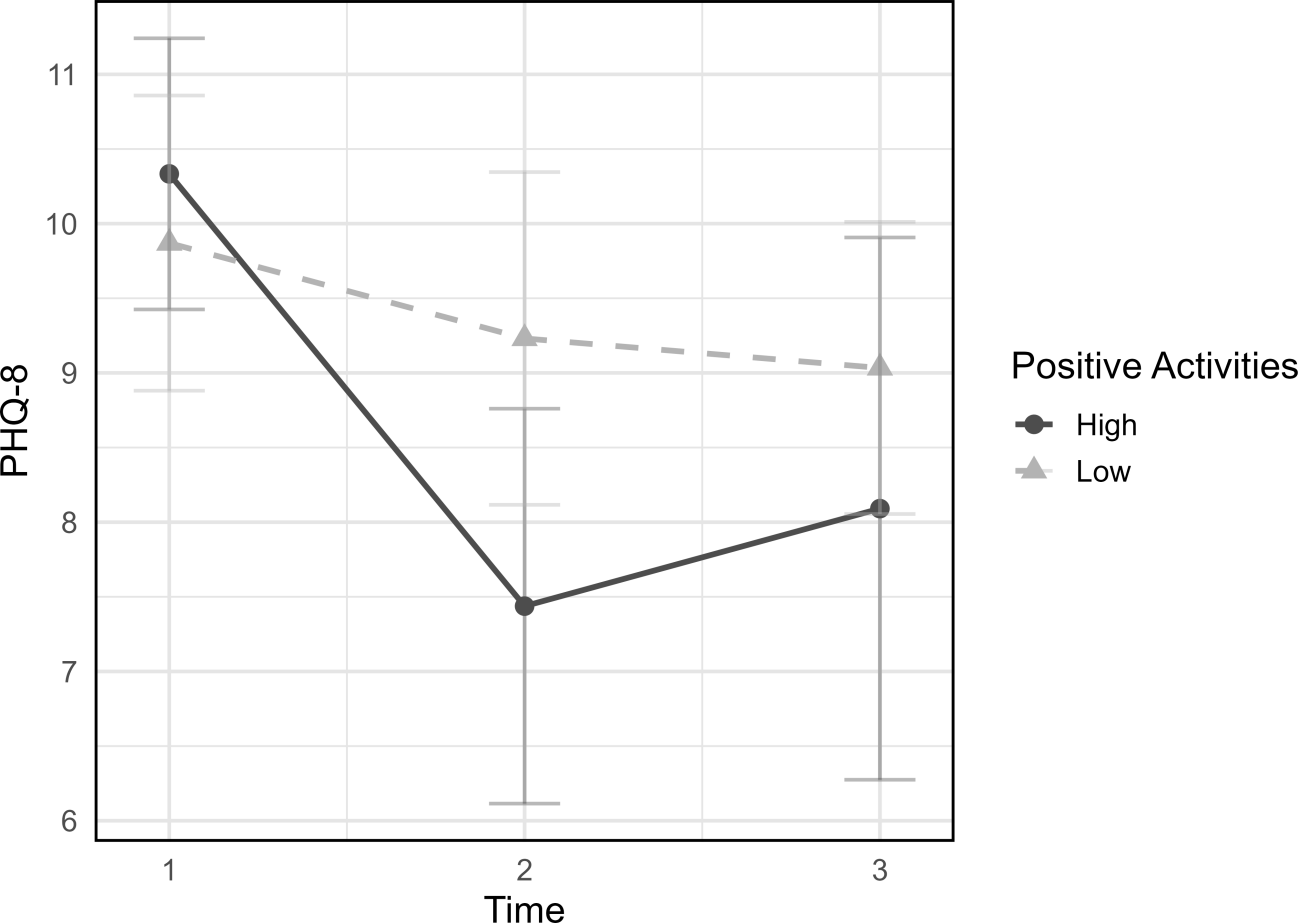
Effects of positive activity engagement on depression over time. Weekly PHQ-8 scores are plotted by high versus low positive activity engagement groups. Positive activity engagement scores were group-mean centered at each time point. High and Low groups were determined by whether the weekly positive activities were above or below the mean at each time point. Participants with higher positive activity engagement exhibited a greater reduction in depressive symptoms over time. PHQ-8, Patient Health Questionnaire-8.

The multilevel model included weekly positive activities, log counts, and time as fixed effects, along with their interactions (Weekly positive activities × Time, Weekly log counts × Time), with random intercepts for participants. Weekly positive activities were divided into high and low groups based on whether the weekly scores were above or below the sample mean at each time point. Multilevel analysis revealed a statistically significant interaction between weekly positive activities and time (B = -0.016, *p* = .011), indicating that individuals who engaged more frequently in positive activities exhibited a steeper decline in depressive symptoms during the intervention period (**Figure 2**). In contrast, the interaction between weekly log counts and time was not significant (*p* = .567), suggesting that weekly log counts alone did not significantly contribute to symptom reduction.

To further examine the relationship between participation and symptom improvement, correlations and regression analyses were conducted between app usage variables and changes in depression scores (PHQ-8, BDI-II, HDRS-17). No significant correlations were identified in the PHQ-8 scores from baseline to post-treatment. In contrast, significant positive correlations were found between positive activities and positive activity scores and changes in BDI-II scores. For HDRS-17 scores, significant positive correlations were observed for all app usage variables. The complete correlation values between depression change scores and app usage variables are presented in **Supplementary Table S5**.

**Figure 3:**
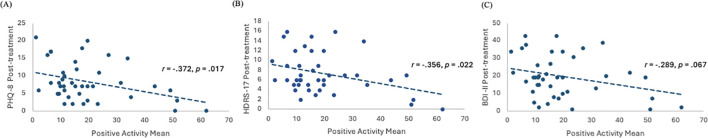
Scatterplots showing the associations between positive activity mean and post-treatment depression scores. Each panel illustrates the relationship between post-treatment depression scores (PHQ-8, HDRS-17, BDI-II) and the mean number of positive activities performed over 7 weeks of app use. The dashed lines represent fitted regression lines.

Additionally, to examine the relationship between positive activity participation and depressive symptoms at the end of the intervention, correlations were calculated between post-treatment depression scores (PHQ-8, BDI-II, HDRS-17) and the mean number of positive activities performed over seven weeks of app use. As shown in **Table 3**, significant negative correlations were observed between mean positive activity and depressive symptoms measured by the PHQ-8 (*r* = –.372, *p* = .017) and HDRS-17 (*r* = –.356, *p* = .022), whereas the correlation with the BDI-II was not significant (*r* = –.289, *p* = .067). These findings indicate that participants who engaged more frequently in positive activities tended to report lower levels of depression at the end of the intervention. Complementing these results, **Figure 3** illustrates the negative associations between mean positive activity scores and post-treatment depression outcomes, with the inverse trend most pronounced for the PHQ-8.

**Table 3:** Correlation between post-treatment depression scores and mean positive activity variable.

	Positive Activity Mean	BDI-II Post-treatment	HDRS-17 Post-treatment	PHQ-8 Post-treatment
Positive Activity Mean	1			
BDI-II Post-treatment	-.289	1		
HDRS-17 Post-treatment	-.356*	.711*	1	
PHQ-8 Post-treatment	-.372*	.916**	.667**	1

**Correlation is significant at the 0.01 level (two-tailed).

*Correlation is significant at the 0.05 level (two-tailed).

Positive Activity Mean refers to the average number of positive activities performed over 7 weeks of app use.

BDI-II, Beck Depression Inventory-II; HDRS-17, 17-item Hamilton Depression Rating Scale; PHQ-8, Patient Health Questionnaire.

Regression analyses further examined the relationship between positive activities and changes in BDI-II scores from baseline to post-treatment. The model was statistically significant (*F* (1, 39) = 5.329, *p* = .026), explaining 12.0% of the variance in BDI-II changes (R² = 0.12). Greater participation in positive activities was significantly associated with a greater reduction in BDI-II scores (B = 0.035, 95% CI: 0.004–0.066; *p* = .026; **Table 4**). For the HDRS-17, a multiple regression analysis including all app usage metrics as predictors was initially significant (*F* (4, 36) = 4.796, *p* = .003). Due to multicollinearity between positive activities and positive activity scores (VIF > 1000), the positive activity score was excluded from the final model. The revised model remained significant (*F* (3, 37) = 5.872, *p* = .002), with positive activities showing a significant positive association (*β* = 1.283, *p* = .003) and total activity count showing a significant negative association (*β* = -1.005, *p* = .020) with HDRS-17 score reductions. The detailed results are presented in **Table 4**.

**Table 4:** Regression analyses predicting depression score changes.

Predictor	BDI-II			HDRS-17			Tolerance	VIF
	B	SE	*p*	B	SE	*p*		
(Constant)	0.021	2.573	.994	3.059	2.117	.157	–	–
Total Login Count	–	–	–	0.072	0.064	.264	0.597	1.676
Total Activity Count	–	–	–	-0.027	0.011	.020	0.107	9.318
Positive Activity Total	0.035	0.015	.026	0.047	0.015	.003	0.115	8.725

BDI-II Change = difference in BDI-II scores (post – baseline). HDRS-17 Change = difference in HDRS-17 scores (post – baseline). Positive Activity Total = number of positive activities completed. SE = Standard Error. Only Positive Activity Total was tested in the BDI-II regression.

BDI-II, Beck Depression Inventory-II; HDRS-17, 17-item Hamilton Depression Rating Scale.

Finally, the relationship between app satisfaction (uMARS) and other key variables (changes in depression scores and app usage indicators) was also examined. Correlation analyses revealed that app satisfaction (uMARS) was not significantly correlated with changes in BDI-II, HDRS-17, PHQ-8, or actual app usage indicators (total login count, total activity count, positive activity total, and positive activity score total). The detailed results of these analyses are presented in **Supplementary Table S5**.

### 3.6 Changes in depression scores by severity group

Changes in depression scores at baseline, Week 4, and post-treatment were further analyzed based on severity groups using repeated-measures ANOVA. For the PHQ-8, BDI-II, and HDRS-17 scores, significant main effects of time were observed, with different change patterns across severity groups (*F* = 10.27, *p* <.001, *F* = 9.857, *p* <.001, and *F* = 67.595, *p* <.001, respectively). For PHQ-8 scores, the severe group showed the largest reduction in PHQ-8 scores, decreasing by 15.50 points from baseline to post-treatment, followed by the moderate group with a decrease of 2.20 points, the mild group with a reduction of 4.50 points, and the normal group with a reduction of 1.53 points. The same patterns were observed for BDI-II scores. The severe group showed the greatest reduction (-8.21 points), followed by the moderate group (-3.16 points), mild group (-4.71 points), and normal group (-1.29 points). For HDRS-17 scores, a comparable trend was found. The severe group showed the greatest reduction (-14.00 points), followed by the moderate group (-8.10 points), the mild group (-6.96 points), and the normal group (-3.00 points). **Supplementary Figure S1** shows the differences in depression score changes across severity groups.

### 3.7 Comparison of depression changes by setting

The effectiveness of the application was also compared between two settings: hospital outpatient clinic and community counseling center. Independent-samples t-tests were conducted to compare changes in depression scores (PHQ-8, BDI-II, and HDRS-17) between the two settings. Although the hospital group demonstrated slightly greater improvements across all depression measures, the differences were not statistically significant (BDI-II: *t* = -0.627, *p* = .267; HDRS-17: *t* = -1.358, *p* = .091; PHQ-8: *t* = -1.304, *p* = .100; **Supplementary Table S6**).

## 4 Discussion

This pilot clinical study offers preliminary evidence for the feasibility and clinical utility of the B-ACT, a culturally adapted digital BA intervention for Korean young adults with depression. Participants demonstrated significant emotional, behavioral, and functional improvements, including reductions in depressive symptoms and anxiety and increases in the level of behavioral activation as well as quality of life. These improvements were evident in both baseline-to-post changes and multilevel analyses confirming significant time effects across the major outcomes.

Beyond these general improvements, participation in emotionally positive activities consistently emerged as the strongest predictor of symptom reduction. In this study, positive emotional activities were defined as activities that participants rated as accompanied by subjectively positive affective states, based on in-app valence ratings collected after each logged activity. Pre–post change analyses revealed that participants who engaged more frequently in such activities showed greater reductions in depressive symptoms, whereas greater total activity volume was associated with smaller clinician-rated symptom reductions (i.e., less improvement), highlighting the importance of emotional quality rather than behavioral quantity. Participants with more severe baseline depression tended to show greater absolute reductions in depressive symptom scores. In addition, comparable outcomes across hospital and community settings suggest the potential scalability of the intervention in diverse care contexts.

The clinical effectiveness of BA in reducing depressive and anxiety symptoms and enhancing the level of behavioral activation is supported by meta-analytic reviews (8, 52–54). Building on this foundation, the present study suggests that these effects may extend to a fully digital format, with additional gains observed in quality of life—particularly in the MCS—and real-world functioning, consistent with literature emphasizing its role in broader psychosocial recovery (55–57). Similar findings have been reported for other digital BA interventions, including MUBS, a personalized BA recommender system; Spark, a digital BA program for adolescents integrating cognitive behavioral and BA strategies; and Moodivate, a fully digital BA intervention for adults, all of which demonstrated feasibility and symptom reduction, with some trials reporting superior remission rates and improvements in anxiety, general health, and fatigue (14, 23, 58). Taken together, these comparisons suggest that B-ACT may have a potential clinical impact comparable to established digital treatments, although this interpretation should be made cautiously given the pilot study design.

The aforementioned digital BA interventions share core BA elements but differ in their approaches to activity structure, mood monitoring, and reinforcement. MUBS (23) combines a large validated activity catalog with machine-learning–based personalization and includes core BA components (planning, logging, reflection, and progress visualization). However, its reinforcement relies on simplified feedback (e.g., thumbs-up/down and a single daily mood score), which limits fine-grained activity–mood monitoring despite strong personalization. Spark (15, 58) delivers a five-week, adolescent-focused program with psychoeducation, activity scheduling, problem-solving, mindfulness, and relapse prevention, supported by gamified rewards and daily mood check-ins, but emotion tracking remains coarse and is not assessed at the per-activity level. Moodivate (14) provides self-guided goal setting and activity scheduling with badge-based reinforcement and brief daily mood ratings, again without fine-grained per-activity affective monitoring. In contrast, B-ACT integrates mandatory per-activity valence logging with coin-based gamification and visual dashboards, enabling more detailed tracking of activity–emotion links while providing structured feedback to support self-monitoring and reflection. This contrast highlights B-ACT’s distinctive emphasis on fine-grained activity–mood monitoring within a digital BA framework. This comparison helps frame the subsequent interpretation of how specific patterns of activity engagement, particularly emotionally positive activities, were associated with clinical improvement in the present study.

In this study, participation in emotionally positive activities appeared to play an important role in digital BA. Higher participation in such activities was associated with reduced depressive symptoms, whereas the total activity count was negatively associated with clinician-rated depressive symptoms, suggesting that frequency alone may not be a sufficient therapeutic target. These findings underscore that in digital BA, the emotional valence of activities may be a more robust predictor of symptom improvement than their sheer number. Prior studies have reported mixed associations between app engagement and clinical improvement, with some showing dose–response effects (15, 59–61) and others reporting null or inverse relationships (62, 63). The present findings may help reconcile these inconsistencies by indicating that participation in positive activities, rather than raw usage metrics, is a more reliable indicator of therapeutic benefit.

This pattern may be partly explained by the flexible activity-tracking system of the B-ACT, in which participants freely selected or entered activities, generating diverse behavioral data. Some activities may not have elicited positive emotional responses and may even reflect avoidance depending on individual readiness or context, which could account for the negative association between total activity count and clinician-rated symptoms. In contrast, positively rated activities consistently predicted clinical improvement. This interpretation aligns with the core theoretical principle of BA that the therapeutic value of a behavior lies not in its frequency but in its functional outcome, particularly whether it produces positive reinforcement (64). The B-ACT operationalizes this principle through personalized feedback highlighting previously effective behaviors, supporting informal self-monitoring and goal setting. Although uMARS-measured user satisfaction was not significantly associated with app usage or symptom reduction, these findings suggest that behavior–emotion alignment may be more critical for clinical benefit than overall app satisfaction. Sustained participation in positive activities was associated with greater symptom reduction over time, consistent with the view that the effects of BA may accumulate gradually rather than emerge immediately. The relatively high retention rate of the B-ACT (87.2%) suggests that engagement with core therapeutic processes can be maintained over the intervention period under structured research conditions.

Gamified elements, real-time feedback, and cumulative tracking can enhance extrinsic engagement by providing immediate rewards and visible progress. Meanwhile, grounding the intervention in BA theory may have fostered intrinsic motivation by reinforcing behaviors that naturally produce positive emotional outcomes (64), promoting a self-sustaining cycle of engagement. Collectively, these mechanisms may have played a role in the observed adherence and symptom improvement in this pilot trial. Although further validation in naturalistic settings is warranted, the findings support the potential of the B-ACT as a scalable digital BA intervention.

Notably, greater absolute symptom reduction was observed among participants with higher baseline depression severity, suggesting potential utility for individuals with moderate-to-severe symptoms. This finding is consistent with the results of a large RCT showing that BA outperformed cognitive therapy in patients with severe depression (65). Similarly, the NICE guidelines recommend BA as the first-line treatment across all severity levels (66). Extending this evidence cautiously to digital delivery, the present pilot findings suggest that B-ACT may also be beneficial among higher-severity users. This pattern is further echoed by Spark, which showed clinical benefits across a broad severity spectrum, including moderate-to-severe cases (58). Together, these results suggest that BA-based digital interventions may be applicable to more clinically burdened populations and help challenge the notion that such tools are only suitable for mild cases.

In this study, comparable symptom improvements were observed across both hospital and community settings, supporting the feasibility of B-ACT in diverse care environments. This consistency suggests that the core components of the intervention are transferable beyond institutional contexts, thereby enhancing scalability. As digital mental health services expand, B-ACT may be well suited for integration into primary care, university counseling centers, and workplace wellness programs, particularly for underserved populations facing structural or logistical barriers to care. This potential is consistent with broader efforts to improve access and equity through digital mental health services (67).

Beyond scalability, systematic cultural adaptation represents another strength of this study. The B-ACT was not a simple translation but was tailored to enhance relevance for Korean young adults through targeted modifications, including localization of the activity catalog, adaptation of psychoeducational content to common stressors (e.g., academic pressure and parent–child conflict), and the use of culturally attuned language and tone. These adaptations were intended to improve ecological validity and user engagement. Consistent with culturally adapted digital interventions in other settings, such as the Chinese Step-by-Step program (68) and the Kuamsha BA app for South African adolescents (69), the present approach underscores the importance of aligning intervention content with local cultural and psychosocial contexts. Given that explicit documentation of cultural adaptation processes in digital interventions remains limited (70), this study contributes to the emerging literature in this area.

Although these findings are encouraging, several limitations should be noted. First, the single-arm, non-randomized design without a control group limited causal inference and may have introduced selection bias due to reliance on a convenience clinical sample primarily recruited from outpatient psychiatric and counseling settings, thereby constraining generalizability. Second, the modest sample size reduced statistical power. Third, the reliance on a sample of young adults with relatively high digital literacy further constrains applicability to older or less technologically adept populations. Fourth, reliance on self-report measures may have introduced response bias, and the short follow-up period precluded assessment of long-term effects. Moreover, the relatively higher remission and response rates based on the clinician-administered HDRS-17 may partly reflect assessor-related bias, given the open-label nature of the study and the lack of rater blinding, particularly at the hospital site where treating psychiatrists conducted clinician-administered assessments. Fifth, our assessment battery did not capture key constructs such as emotional dysregulation, impulsivity, and cognitive-behavioral beliefs/values, thereby limiting mechanistic interpretation. Finally, although gamified features were incorporated to promote engagement, their independent contribution to clinical outcomes remains uncertain. In addition, because this was a single-arm study in which most participants were receiving ongoing pharmacotherapy and had repeated face-to-face contact with assessors, the observed symptom changes cannot be attributed solely to the digital intervention. The potential contribution of medication effects and nonspecific therapeutic factors, including in-person interactions, should therefore be considered. Future studies should employ randomized controlled designs with active comparators, stratify participants by baseline severity, extend follow-up to evaluate maintenance of effects, and include broader non-clinical populations to enhance generalizability. Moreover, systematic evaluation of gamification components, alongside broader psychological constructs such as emotion regulation, impulsivity, and cognitive-behavioral beliefs/values, will be essential for a more comprehensive understanding of therapeutic mechanisms.

Despite these limitations, this study also has notable strengths. B-ACT is grounded in core BA principles and delivered through structured weekly modules, representing the first clinical evaluation of a culturally adapted BA app for Korean young adults. The use of multilevel modeling enhanced analytical precision, and medication stability helped reduce potential confounding. Importantly, this study addresses a growing public health need related to depression and social isolation among Korean youth. By delivering BA via a mobile platform attuned to users’ digital and psychosocial contexts, B-ACT demonstrates promise as a culturally responsive and scalable intervention.

## 5 Conclusion

This study provides preliminary evidence for the utility of B-ACT, a culturally adapted digital BA intervention for young adults with depression. Participants showed statistically significant baseline-to-post improvements in depressive symptoms, anxiety, the level of behavioral activation, and quality of life. Engagement in emotionally positive activities, rather than overall activity volume, emerged as the most consistent predictor of symptom reduction. The intervention was feasible in both hospital and community settings, supporting its feasibility for broader implementation. Future research should evaluate long-term outcomes and confirm causal effects through randomized controlled trials.

## Data Availability

The original contributions presented in the study are included in the article/**Supplementary Material**. Further inquiries can be directed to the corresponding author/s.

## Supplementary material

Supplementary Figure 1 Changes in depression scores by severity group across time points for (1) PHQ-8, (B) BDI-II, and (C) HDRS-17. Participants were categorized into four severity groups based on baseline scores: Normal, Mild, Moderate, and Severe. The cut-off criteria were as follows—PHQ-8: Normal (≤9), Mild (10–14), Moderate (15–19), Severe (≥20); BDI-II: Normal (≤13), Mild (14–19), Moderate (20–28), Severe (≥29); HDRS-17: Normal (≤7), Mild (8–16), Moderate (17–23), Severe (≥24). Across all three measures, depressive symptoms decreased over the 8-week period, with the most substantial reductions observed in the Severe group. Scores in the Normal group remained relatively stable throughout. Abbreviations: PHQ-8, Patient Health Questionnaire-8; BDI-II, Beck Depression Inventory-II; HDRS-17, 17-item Hamilton Depression Rating Scale
