# Supplementary figures and images for: A behavioral activation mobile application for depression among Korean young adults: a pilot study of multi-modal app usage patterns and clinical outcomes
Source: Front Psychiatry. 2026 Jan 22;16:1707034. doi: 10.3389/fpsyt.2025.1707034 (PMC12872826; doi:10.3389/fpsyt.2025.1707034)

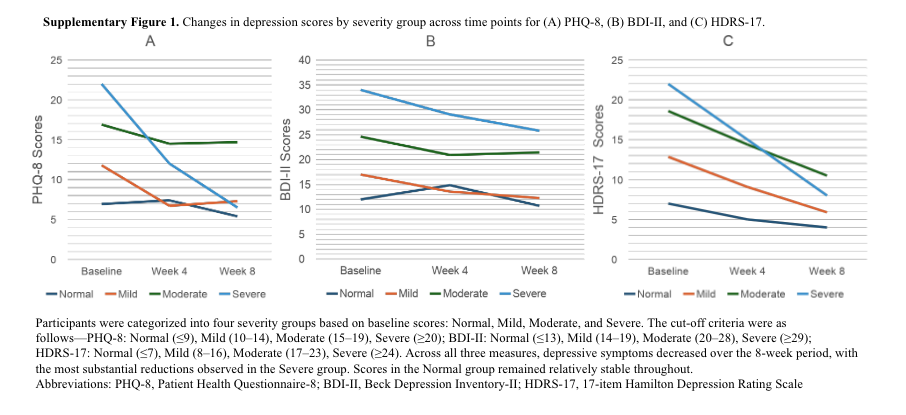

Supplement: Supplementary Figure 1 — Changes in depression scores by severity group across time points for (1) PHQ-8, (B) BDI-II, and (C) HDRS-17. Participants were categorized into four severity groups based on baseline scores: Normal, Mild, Moderate, and Severe. The cut-off criteria were as follows—PHQ-8: Normal (≤9), Mild (10–14), Moderate (15–19), Severe (≥20); BDI-II: Normal (≤13), Mild (14–19), Moderate (20–28), Severe (≥29); HDRS-17: Normal (≤7), Mild (8–16), Moderate (17–23), Severe (≥24). Across all three measures, depressive symptoms decreased over the 8-week period, with the most substantial reductions observed in the Severe group. Scores in the Normal group remained relatively stable throughout. Abbreviations: PHQ-8, Patient Health Questionnaire-8; BDI-II, Beck Depression Inventory-II; HDRS-17, 17-item Hamilton Depression Rating Scale [file Image1.png]
