# Supplementary material for: A behavioral activation mobile application for depression among Korean young adults: a pilot study of multi-modal app usage patterns and clinical outcomes
Source: Front Psychiatry. 2026 Jan 22;16:1707034. doi: 10.3389/fpsyt.2025.1707034 (PMC12872826; doi:10.3389/fpsyt.2025.1707034)
Supplement: Supplementary file 2 [file Table1.docx]

**Supplementary Table 1**. Weekly Module Themes and Psychoeducational Video Content in the 7-Week B-ACT Program

| **Week** | **Module Theme** | **Psychoeducational video** |
| --- | --- | --- |
| **1** | Understanding Depression and Behavioral Activation | Tracking Your Mood Over the Week |
|  |  | What Is Depression? |
|  |  | Why Does Depression Occur? |
|  |  | What Is Behavioral Activation? |
|  |  | Understanding the Link Between Activities and Mood |
|  |  | How Does Behavioral Activation Help Improve Mood? |
|  |  | Understanding Avoidance in Depression |
|  |  | Frequently Asked Questions About Antidepressant Medication |
| **2** | Understanding the Relationship Between Activities and Mood & the Importance of Core Daily Routines | What Are Core Daily Activities? |
|  |  | Avoidance Based on Mood vs. Value-Based Goal Setting |
| **3** | Identifying Personal Values and Planning Value-Based Activities | Exploring Life Values Across Different Domains |
|  |  | How to Set Goals Based on Personal Values |
| **4** | Setting Rewards for Achieving Goal Activities | How to Reward Yourself After Completing Goal Activities |
| **5** | Practicing Problem-Solving Skills | What Is the COPE Model for Problem-Solving? |
| **6** | Practicing Mindfulness Skills | Understanding Rumination and Mindfulness |
|  |  | When and How to Use Mindfulness Skills |
| **7** | Review and Relapse Prevention | Managing Future Episodes of Depression with Behavioral Activation |

Abbreviations: COPE; Clarify, generate Options, Perform, Evaluate
