# Supplementary material for: A behavioral activation mobile application for depression among Korean young adults: a pilot study of multi-modal app usage patterns and clinical outcomes
Source: Front Psychiatry. 2026 Jan 22;16:1707034. doi: 10.3389/fpsyt.2025.1707034 (PMC12872826; doi:10.3389/fpsyt.2025.1707034)
Supplement: Supplementary file 3 [file Table2.docx]

**Supplementary Table 2.** Medication regime at baseline

| **Type and subtype of medication** | **All**  **(n=47)** | **Hospital Outpatient**  **(n=25/25)** | **Community counseling center**  **(n=10/22)** |
| --- | --- | --- | --- |
| **Antidepressants** |  |  |  |
| None | 14 | 1 | 13 |
| SSRI | 14 | 8 | 6 |
| SNRI | 7 | 6 | 1 |
| NDRI | 0 | 0 | 0 |
| NaSSA | 0 | 0 | 0 |
| Other AD | 2 | 1 | 1 |
| Combination of ADs | 10 | 9 | 1 |
| **Antipsychotics** |  |  |  |
| None | 29 | 8 | 21 |
| AP | 13 | 12 | 1 |
| Combination of APs | 5 | 5 | 0 |

Numbers represent the number of individuals prescribed each type of medication at baseline.

*n* = number of participants currently taking medication / total number of participants in each setting.

Abbreviations: AD, Antidepressants; AP, Antipsychotics; SSRI, Selective serotonin reuptake inhibitor; SNRI, Serotonin-norepinephrine reuptake inhibitor; NDRI, Norepinephrine-dopamine reuptake inhibitor; NaSSA, Noradrenergic and specific serotonergic antidepressant.
