# Supplementary material for: A behavioral activation mobile application for depression among Korean young adults: a pilot study of multi-modal app usage patterns and clinical outcomes
Source: Front Psychiatry. 2026 Jan 22;16:1707034. doi: 10.3389/fpsyt.2025.1707034 (PMC12872826; doi:10.3389/fpsyt.2025.1707034)
Supplement: Supplementary file 4 [file Table3.docx]

**Supplementary Table 3.** Correlation Matrix of Weekly Changes in PHQ-8 Scores and Positive Activity Counts

|  | 1 | 2 | 3 | 4 | 5 | 6 | 7 | 8 | 9 | 10 | 11 | 12 | 13 | 14 |
| --- | --- | --- | --- | --- | --- | --- | --- | --- | --- | --- | --- | --- | --- | --- |
| 1. PHQ-8 Change 1 | 1 |  |  |  |  |  |  |  |  |  |  |  |  |  |
| 1. PHQ-8 Change 2 | -.405^**^ | 1 |  |  |  |  |  |  |  |  |  |  |  |  |
| 1. PHQ-8 Change 3 | 0.036 | -.386^*^ | 1 |  |  |  |  |  |  |  |  |  |  |  |
| 1. PHQ-8 Change 4 | -0.167 | 0.279 | -.520^**^ | 1 |  |  |  |  |  |  |  |  |  |  |
| 1. PHQ-8 Change 5 | 0.106 | -0.237 | 0.288 | -.508^**^ | 1 |  |  |  |  |  |  |  |  |  |
| 1. PHQ-8 Change 6 | -0.11 | -.370^*^ | 0.228 | -0.192 | -0.136 | 1 |  |  |  |  |  |  |  |  |
| 1. PHQ-8 Change 7 | 0.042 | -0.203 | -0.012 | -0.189 | 0.161 | -0.205 | 1 |  |  |  |  |  |  |  |
| 1. Positive Activity W1 | -0.151 | 0.115 | 0.03 | 0.265 | 0.043 | -0.039 | -0.035 | 1 |  |  |  |  |  |  |
| 1. Positive Activity W2 | -0.135 | -0.107 | 0.073 | 0.135 | 0.1 | 0.112 | -0.05 | .527^**^ | 1 |  |  |  |  |  |
| 1. Positive Activity W3 | -0.187 | -0.057 | 0.224 | 0.073 | 0.076 | 0.27 | 0.039 | .636^**^ | .733^**^ | 1 |  |  |  |  |
| 1. Positive Activity W4 | -0.046 | -0.159 | 0.155 | 0.111 | 0.074 | 0.132 | 0.047 | .576^**^ | .741^**^ | .615^**^ | 1 |  |  |  |
| 1. Positive Activity W5 | -0.233 | 0.126 | 0.011 | 0.007 | 0.192 | 0.156 | -0.136 | .532^**^ | .726^**^ | .728^**^ | .525^**^ | 1 |  |  |
| 1. Positive Activity W6 | -0.106 | -0.064 | -0.1 | 0.089 | 0.104 | 0.185 | 0.032 | .433^**^ | .576^**^ | .651^**^ | .378^*^ | .728^**^ | 1 |  |
| 1. Positive Activity W7 | -0.113 | -0.028 | 0.14 | 0.018 | 0.141 | 0.149 | 0.033 | .522^**^ | .681^**^ | .775^**^ | .504^**^ | .797^**^ | .817^**^ | 1 |

**. Correlation is significant at the 0.01 level (two-tailed).

*. Correlation is significant at the 0.05 level (two-tailed).

PHQ-8 Change X refers to the change in PHQ-8 scores between consecutive weeks (e.g., PHQ-8 Change 1 indicates the change between Week 1 and Week 2). Positive Activity WX represents the number of positive activities performed in each week (e.g., Positive Activity W1 is the number of positive activities in Week 1).

Abbreviations: PHQ-8, Patient Health Questionnaire-8
