# Supplementary material for: A behavioral activation mobile application for depression among Korean young adults: a pilot study of multi-modal app usage patterns and clinical outcomes
Source: Front Psychiatry. 2026 Jan 22;16:1707034. doi: 10.3389/fpsyt.2025.1707034 (PMC12872826; doi:10.3389/fpsyt.2025.1707034)
Supplement: Supplementary file 5 [file Table4.docx]

**Supplementary Table 4**. Correlation Matrix of Weekly Changes in PHQ-8 Scores and Changes in Positive Activities

|  | **1** | **2** | **3** | **4** | **5** | **6** | **7** | **8** | **9** | **10** | **11** | **12** | **13** |
| --- | --- | --- | --- | --- | --- | --- | --- | --- | --- | --- | --- | --- | --- |
| **1. PHQ-8 Change 1** | 1 |  |  |  |  |  |  |  |  |  |  |  |  |
| **2. PHQ-8 Change 2** | -.405^**^ | 1 |  |  |  |  |  |  |  |  |  |  |  |
| **3. PHQ-8 Change 3** | 0.036 | -.386^*^ | 1 |  |  |  |  |  |  |  |  |  |  |
| **4. PHQ-8 Change 4** | -0.167 | 0.279 | -.520^**^ | 1 |  |  |  |  |  |  |  |  |  |
| **5. PHQ-8 Change 5** | 0.106 | -0.237 | 0.288 | -.508^**^ | 1 |  |  |  |  |  |  |  |  |
| **6. PHQ-8 Change 6** | -0.11 | -.370^*^ | 0.228 | -0.192 | -0.136 | 1 |  |  |  |  |  |  |  |
| **7. PHQ-8 Change 7** | 0.042 | -0.203 | -0.012 | -0.189 | 0.161 | -0.205 | 1 |  |  |  |  |  |  |
| **8. Positive Activity Change 1** | 0.005 | 0.225 | -0.053 | 0.092 | -0.07 | -0.157 | 0.023 | 1 |  |  |  |  |  |
| **9. Positive Activity Change 2** | 0.066 | -0.072 | -0.193 | 0.101 | 0.044 | -0.211 | -0.128 | -.556^**^ | 1 |  |  |  |  |
| **10. Positive Activity Change 3** | -0.158 | 0.111 | 0.055 | -0.059 | -0.009 | 0.135 | -0.017 | 0.084 | -.593^**^ | 1 |  |  |  |
| **11. Positive Activity Change 4** | 0.151 | -0.294 | 0.172 | 0.118 | -0.089 | 0.009 | 0.173 | -0.044 | 0.156 | -.731^**^ | 1 |  |  |
| **12. Positive Activity Change 5** | -0.124 | 0.243 | 0.158 | -0.119 | 0.078 | -0.081 | -0.206 | -0.027 | 0.153 | -0.182 | -0.035 | 1 |  |
| **13. Positive Activity Change 6** | 0.074 | -0.023 | -.342^*^ | 0.065 | -0.122 | -0.049 | -0.021 | 0.195 | 0.056 | -0.112 | -0.001 | -0.236 | 1 |

**. Correlation is significant at the 0.01 level (two-tailed).

*. Correlation is significant at the 0.05 level (two-tailed).

PHQ-8 Change X refers to the change in PHQ-8 scores between consecutive weeks (e.g., PHQ-8 Change 1 indicates the change between Week 1 and Week 2). Positive Activity Change X refers to the change in the number of positive activities between consecutive weeks (e.g., Positive Activity Change 1 indicates the change between Week 1 and Week 2).

Abbreviations: PHQ-8, Patient Health Questionnaire-8
