# Supplementary material for: A behavioral activation mobile application for depression among Korean young adults: a pilot study of multi-modal app usage patterns and clinical outcomes
Source: Front Psychiatry. 2026 Jan 22;16:1707034. doi: 10.3389/fpsyt.2025.1707034 (PMC12872826; doi:10.3389/fpsyt.2025.1707034)
Supplement: Supplementary file 6 [file Table5.docx]

**Supplementary Table 5**. Correlation Matrix of App Satisfaction (uMARS), Depression Score Changes, and App Usage Variables

|  | 1 | 2 | 3 | 4 | 5 | 6 | 7 | 8 |
| --- | --- | --- | --- | --- | --- | --- | --- | --- |
| 1. uMARS Total | 1 |  |  |  |  |  |  |  |
| 1. BDI-II Changes | -0.092 | 1 |  |  |  |  |  |  |
| 1. HDRS-17 Changes | 0.187 | .652^**^ | 1 |  |  |  |  |  |
| 1. PHQ-8 Changes | -0.1 | .794^**^ | .555^**^ | 1 |  |  |  |  |
| 1. Total Login Count | 0.039 | 0.15 | .333^*^ | 0.112 | 1 |  |  |  |
| 1. Total Activity Count | 0.183 | 0.225 | .328^*^ | 0.125 | .624^**^ | 1 |  |  |
| 1. Positive Activity Total | 0.208 | .347^*^ | .457^**^ | 0.209 | .587^**^ | .942^**^ | 1 |  |
| 1. Positive Activity Score Total | 0.209 | .348^*^ | .463^**^ | 0.211 | .587^**^ | .940^**^ | 1.000^**^ | 1 |

**. Correlation is significant at the 0.01 level (two-tailed)
*. Correlation is significant at the 0.05 level (two-tailed)

BDI-II Change, HDRS-17 Change, PHQ-8 Change refer to the change in scores from baseline to post-treatment, Total Login Count represents the total number of app logins, Total Activity Count indicates the total number of activities performed, Positive Activity Total is the total number of positive activities performed. Positive Activity Score Total is the total score of positive activities.

Abbreviations: uMARS, User Version of the Mobile Application Rating Scale; BDI-II, Beck Depression Inventory-II; HDRS-17, 17-item Hamilton Depression Rating Scale; PHQ-8, Patient Health Questionnaire-8
