# Supplementary material for: A behavioral activation mobile application for depression among Korean young adults: a pilot study of multi-modal app usage patterns and clinical outcomes
Source: Front Psychiatry. 2026 Jan 22;16:1707034. doi: 10.3389/fpsyt.2025.1707034 (PMC12872826; doi:10.3389/fpsyt.2025.1707034)
Supplement: Supplementary file 7 [file Table6.docx]

**Supplementary Table 6**. Comparison of Depression Changes by Setting

| **Measure** | **Hospital (N=21)** | | **Community (N=20)** | | ***t*** | ***p*** | **Cohen's *d*** |
| --- | --- | --- | --- | --- | --- | --- | --- |
|  | ***M*** | **SD** | ***M*** | **SD** |  |  |  |
| **BDI-II Change** | 5.8095 | 9.74997 | 3.8 | 10.76349 | -0.627 | 0.267 | 10.25625 |
| **HDRS-17 Change** | 7.8095 | 3.58635 | 6.25 | 3.76794 | -1.358 | 0.091 | 3.67594 |
| **PHQ-8 Change** | 3.9048 | 4.40346 | 2.0 | 4.94177 | -1.304 | 0.1 | 4.67346 |

BDI-II Change, HDRS-17 Change, PHQ-8 Change refer to the change in scores from baseline to post-treatment

Abbreviations: BDI-II, Beck Depression Inventory-II; HDRS-17, 17-item Hamilton Depression Rating Scale; PHQ-8, Patient Health Questionnaire-8
